# Enzymatic synthesis of high-titer nicotinamide mononucleotide with a new nicotinamide riboside kinase and an efficient ATP regeneration system

**DOI:** 10.1186/s40643-022-00514-6

**Published:** 2022-03-21

**Authors:** Xiao-Long Qian, Yi-Si Dai, Chun-Xiu Li, Jiang Pan, Jian-He Xu, Bozhong Mu

**Affiliations:** 1 State Key Laboratory of Bioreactor Engineering, East China University of Science and Technology, Shanghai, 200237 People’s Republic of China; 2 Shanghai Collaborative Innovation Center for Biomanufacturing, East China University of Science and Technology, 130 Meilong Road, Shanghai, 200237 People’s Republic of China; 3 Suzhou Bioforany EnzyTech Co. Ltd, No. 8 Yanjiuyuan Road, Economic Development Zone, Changshu, Jiangsu 215512 People’s Republic of China

**Keywords:** Enzymatic phosphorylation, Nicotinamide riboside, Nicotinamide riboside kinase, β-Nicotinamide mononucleotide (NMN), ATP regeneration system

## Abstract

**Background:**

β-Nicotinamide mononucleotide (NMN) is the direct precursor of nicotinamide coenzymes such as NAD^+^ and NADP^+^, which are widely applied in industrial biocatalysis especially involving cofactor-dependent oxidoreductases. Moreover, NMN is a promising candidate for medical uses since it is considered to be beneficial for improving health of aged people who usually suffer from an insufficient level of NAD^+^. To date, various methods have been developed for the synthesis of NMN. Chemical phosphorylation of nicotinamide riboside (NR) to NMN depends on excessive phosphine oxychloride and delicate temperature control, while fermentation of NMN is limited by low product titers, making it unsuitable for industrial-scale NMN production. As a result, the more efficient synthesis process of NMN is still challenging.

**Aim:**

This work attempted to construct an eco-friendly and cost-effective biocatalytic process for transforming the chemically synthesized NR into the highly value-added NMN.

**Results:**

A new nicotinamide riboside kinase (*Klm-*NRK) was identified from *Kluyveromyces marxianus*. The specific activity of purified *Klm-*NRK was 7.9 U·mg^–1^ protein, ranking the highest record among the reported NRKs. The optimal pH of *Klm*-NRK was 7.0 in potassium phosphate buffer. The purified *Klm*-NRK retained a half activity after 7.29 h at 50 °C. The catalytic efficiencies (*k*_cat_/*K*_M_) toward ATP and nicotinamide riboside (NR) were 57.4 s^−1^·mM^−1^ and 84.4 s^−1^·mM^−1^, respectively. In the presence of an external ATP regeneration system (AcK/AcP), as much as 100 g·L^–1^ of NR could be completely phosphorylated to NMN in 8 h with *Klm*-NRK, achieving a molar isolation yield of 84.2% and a space–time yield of 281 g·L^−1^·day^−1^. These inspiring results indicated that *Klm*-NRK is a promising biocatalyst which provides an efficient approach for the bio-manufacturing of NMN in a high titer.

**Graphical Abstract:**

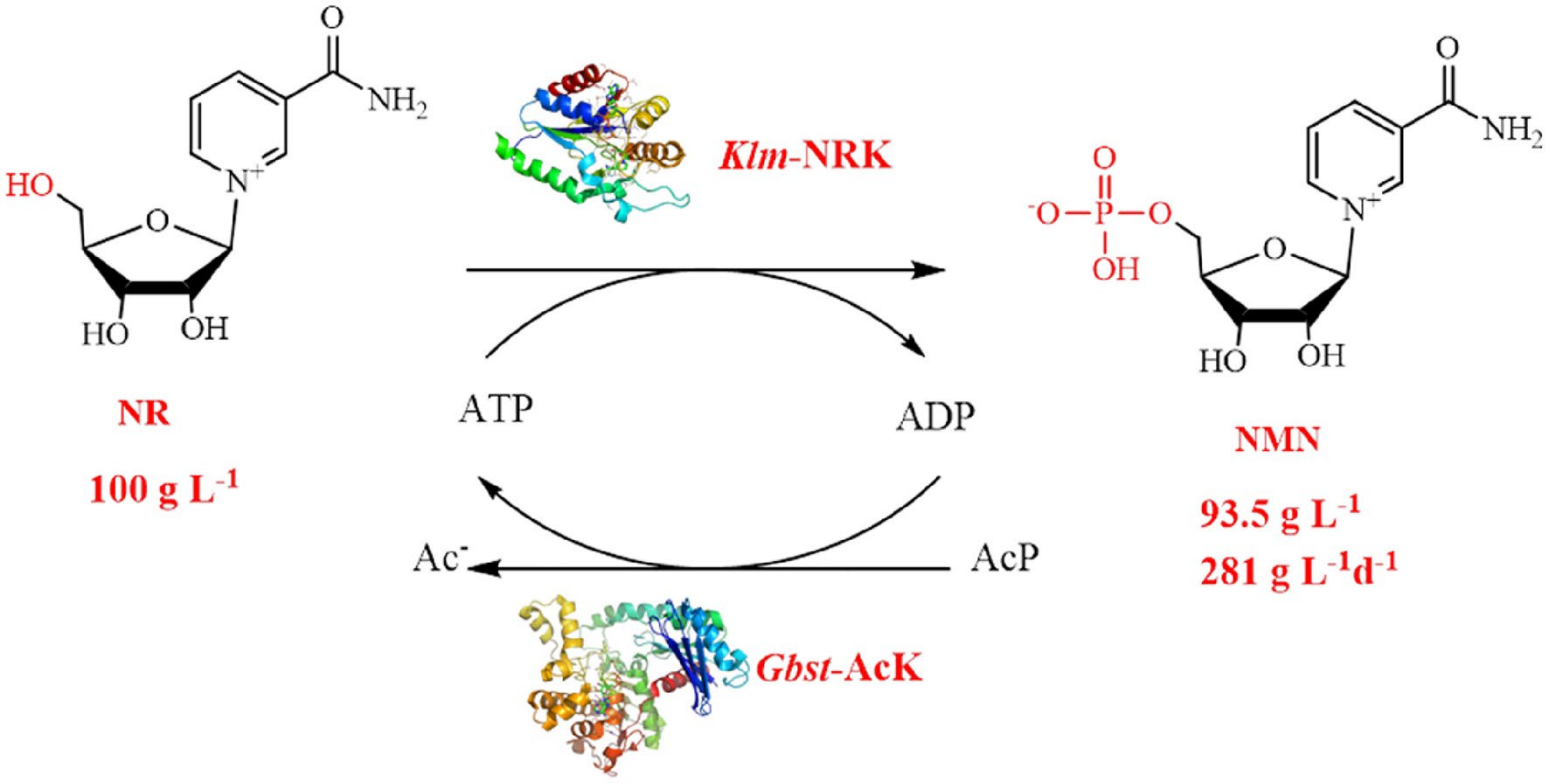

**Supplementary Information:**

The online version contains supplementary material available at 10.1186/s40643-022-00514-6.

## Introduction

β-Nicotinamide mononucleotide (NMN) is an important natural compound which can be found in every living organism. NMN has attracted much attentions as the direct precursor of NAD^+^ and NADP^+^, which are natural coenzymes playing vital roles in in vivo redox reactions (Hong et al. 2020; Yoshino et al. 2018; Ziegler and Nikiforov, 2020). It was suggested that NMN supplementation could compensate for the deficiency of NAD^+^ or NADP^+^, and recent studies have shown that NMN has diverse therapeutic applications such as treatment of neurological disorders, diabetes, obesity and anti-aging (Kiss et al. 2019; Uddin et al. 2017; Yao et al. 2017; Yoshino et al. 2011).

In addition to its potential medical applications, NMN is also very useful in biocatalysis and biotransformation, since NMN can be directly converted into NAD^+^ by coupling with ATP and adenylyl transferases, and further into NADP^+^ after additional phosphorylation (Walt et al. 1984). The addition of NAD^+^/NADP^+^ to reaction system will significantly improve the catalytic performance of in vitro enzymatic redox reactions (Wu et al. 2013; Mordhorst and Andexer 2020). Furthermore, recent studies have shown that NMN can directly participate in redox reactions as a “noncanonical cofactor”. An enoate reductase XenA from *P. putida* can directly use NMN as a coenzyme. When coupled with a glucose dehydrogenase variant, the total turnover number (TTN) of NMN could reach 39,000 (Black et al. 2020a, b; Richardson et al. 2020). Various designing principles and engineering methods were also developed to alter the preference of enzymes toward NMN (King et al. 2020). Considering the importance of NMN, efficient manufacture of NMN at an acceptable cost is vital for the application of NMN in medical and biotechnological industries.

Chemical synthesis of NMN requires the synthesis of nicotinamide riboside (NR) first, usually starting from ribose derivatives and nicotinic acid derivatives (Makarov and Migaud 2019). Chemically synthesized product of nicotinamide riboside usually contains two stereochemical configurations, alpha and beta, while only the beta-isomer has biological activity (Franchetti et al. 2004). The most efficient record is the two-step method reported by Zhang group in 2017, where a stereoselective *N*-glycosylation reaction was mediated by trimethylsilyl trifluoromethanesulfonate (TMSOTf) between the starting materials ethyl nicotinate and 1,2,3,5-tetra-*O*-acetyl-β-d-ribofuranose. After deprotection in ammonia, only beta-isomer was obtained with 85% yield (Zhang and Sauve 2017). Excessive phosphine oxychloride and delicate temperature control (0–5 °C) were required for the subsequent phosphorylation to yield β-NMN (Lee et al. 1999). A series of enzymatic or fermentation methods were also developed for the preparation of NMN, however, either the low product titers or relatively high operation costs hindered their applications (Black et al. 2020a, b; Jeck et al. 1974; Marinescu et al. 2018). In 2021, using metabolic engineering methods, a recombinant *Escherichia coli* strain was constructed by Shoji and co-workers (Shoji et al. 2021), which could produce 6.79 g·L^–1^ of NMN extracellularly from glucose and nicotinic acid, representing the highest record reported so far among the biological methods.

Enzymatic phosphorylation reactions require a phosphoryl donor, and ATP is the most commonly used one. However, the cost of ATP is high, making it unacceptable to be directly added at stoichiometric amount to the reaction system. Besides that, removal of coproduct ADP also complicates the downstream isolation process. Hence, an efficient and economic ATP regeneration system is prerequisite for industrial application of ATP-dependent enzymatic phosphorylation reactions. Several different ATP regeneration systems with different phosphate donors have been developed (Chen and Zhang 2021), such as pyruvate kinase (PK; EC 2.7.1.40) with phosphoenolpyruvate (PEP), creatine kinase (CK; EC 2.7.3.2) with creatine phosphate (CP), acetate kinase (AcK; EC 2.7.2.1) with acetyl phosphate (AcP), and polyphosphate kinase (PPK; EC 2.7.4.1) with polyphosphate (polyP). However, the costs of PEP and CP are so high that limit the applications of PK and CK mediated ATP regeneration systems (Caschera and Noireaux 2015). With regard to PPK/polyP regeneration system, although polyphosphate can be simply obtained from commercial sources or just prepared by heating of NaH_2_PO_4_/Na_2_HPO_4_ mixture in an electric furnace (Honda et al. 2016), the specific activity of PPKs is usually low (less than 30 U/mg), which hindered their practical applications (Achbergerová and Nahálka 2014; Akiyama et al. 1992; Motomura et al. 2014). Besides that, the potentially inhibitory effect of polyP should also be taken into consideration (Zhang et al. 2017). Consequently, AcK/AcP regeneration system, consisting of cheap substrate (Crans and Whitesides 1983) and highly efficient enzyme (Nakajima et al. 1978), is generally regarded as a promising candidate for practical applications.

In this study, we identified a new and highly active nicotinamide riboside kinase (EC 2.7.1.173) from *Kluyveromyces marxianus*. A biocatalytic phosphorylation of NR was developed for the efficient synthesis of NMN. As much as 100 g·L^–1^ of NR was smoothly converted into NMN, by coupling with the economically viable AcK/AcP regeneration system. This approach does not require excessive phosphorus oxychloride, and achieves a higher product titer than other biological methods, rendering it a promising method for the bioproduction of NMN.

## Experimental

### Plasmid, strain, reagents and media

The *Klm*-nrk gene (uniport accession number: W0TD38) and *Gbst*-AcK gene (uniport accession number: A0A0K9HLE0, Nakajima et al. 1978) were optimized according to *E. coli* codon usage alphabet and chemically synthesized by Genscript Co. Ltd. (Nanjing, China). The *Klm*-*nrk* and *Gbst*-*ack* gene fragments were inserted between *Nde*I and *Xho*I of pET21a without stop codon, resulting in recombinant pET21a/*Klm*-*nrk* and pET21a/*Gbst-ack*, and the inserted genes were sequenced by Sanger's DNA sequencing method. *E. coli* BL21(DE3) was used for expression of the recombinant proteins. Nicotinamide riboside chloride and nicotinamide mononucleotide with a high purity (> 97%) were supplied by Furuipharma Co. Ltd. Acetyl phosphate (AcP) was chemically synthesized from 85% phosphoric acid and acetic anhydride (Crans and Whitesides 1983). All the other chemicals of analytical grade were obtained from commercial sources.

Terrific Broth (TB) medium, consisted of 12 g·L^–1^ yeast extract, 24 g·L^–1^ tryptone, 4 mL·L^–1^ glycerol, 9.4 g·L^–1^ K_2_HPO_4_ and 2.2 g·L^–1^ KH_2_PO_4_, was used for the cell growth and protein expression.

### Expression and purification of kinase *Klm-*NRK

Plasmid pET21a/*Klm*-*nrk* was transformed into chemically competent *E. coli* BL21(DE3) cells by heat shock method. Recombinant strains were grown at 37 °C in TB medium containing 100 μg·mL^–1^ ampicillin. When OD_600_ of the culture reached 0.6, the temperature was switched to 25 °C, and IPTG was added to a final concentration of 0.25 mM for induction expression for 12 h. Cells were harvested by centrifugation and stored at –20 °C for further use.

For the purpose of protein purification, recombinant cells were resuspended in buffer A (20 mM sodium phosphate buffer, pH 7.4, 0.5 M NaCl, 20 mM imidazole) and disrupted by ultrasonication. After centrifugation (10,000 × *g*, 30 min) at 4 °C, the supernatant was loaded onto a HisTrap Ni–NTA FF column (5 mL, GE Healthcare Co.) pre-equilibrated with buffer A. Buffer B (20 mM sodium phosphate buffer, pH 7.4, 0.5 M NaCl, 500 mM imidazole) was used for gradient elution of *Klm-*NRK. The purification was then carried out according to the protocol provided by GE Inc. The purity of the collected fractions was examined by SDS-PAGE. The fractions containing the target protein were combined and the elution buffer was replaced with storage buffer (50 mM Tris–HCl, pH 7.4; 25 mM KCl, 0.1 mM EDTA, 2 mM DTT and 50% (v/v) glycerol).

### Enzyme assay and characterization of recombinant *Klm*-NRK

#### Enzyme assay

The coproduct of enzymatic phosphorylation of NR with ATP is ADP, which can be regenerated by PK/PEP with the formation of pyruvate, then pyruvate can be reduced to lactate with the oxidation of NADH catalyzed by LDH. The activity of *Klm*-NRK was assayed spectrophotometrically at 30 °C by monitoring the oxidation of NADH at 340 nm (Dölle and Ziegler, 2009). The standard assay mixture (1 mL) composed of 100 mM potassium phosphate buffer (pH 7.0), 0.5 mM NR, 0.5 mM ATP, 2 mM MgCl_2_, 5 mM phosphoenolpyruvate, 0.15 mM NADH, 3.5 U PK /5 U LDH (pyruvate kinase/lactate dehydrogenase mix, Sigma) and appropriate amount of purified *Klm*-NRK. One unit of *Klm*-NRK activity was defined as the amount of enzyme catalyzing the oxidation of 1 μmol NADH per minute under above conditions.

#### Kinetic analysis

The kinetic parameters of the purified *Klm*-NRK were determined at 30 °C in 50 mM pH 7.5 Tris–HCl containing 50 mM NaCl, 50 mM KCl, 12 mM MgCl_2_ and 0.01% bovine serum albumin, by assaying the initial reaction rates (in triplicate) with varied concentrations of NR (0–0.5 mM, final concentration) and 0.5 mM ATP. For determination of the apparent *K*_M_ value toward ATP, a fixed concentration of NR (0.5 mM) was used (Dölle and Ziegler 2009). For each 1-mL reaction, 6.18 μg of purified *Klm*-NRK was added. The maximal reaction rate (*V*_max_) and apparent Michaelis–Menten constant (*K*_M_) of the purified *Klm*-NRK were calculated by GraphPad Prism 7. The protein concentration was measured by the standard Bradford assay (Bradford, 1976).

#### pH and temperature optima and thermostability

The optimum pH of *Klm*-NRK was determined at 30 °C in the following buffers (0.1 M): sodium citrate (pH 4.0–6.0), sodium phosphate (pH 6.0–8.5), and glycine–NaOH (pH 8.5–1.0). The temperature optimum was determined under the standard condition except for incubated at various temperatures (25–65 °C) for a period of 2 min. Thermal stability was determined by pre-incubating the purified enzyme (2 mg·mL^–1^) at desired inactivation temperatures (30, 40, 50 or 60 °C) for a proper period of time followed by measuring the residual activity. The residual activity was expressed as a percentage of the initial activity (*V*·*V*_0_^–1^). The inactivation rate constants (*k*_D_) were calculated from the slopes of semi-logarithmic plot of residual activity *versus* time (Ln (*V*·*V*_0_^–1^) =—*k*_D_·t). And the half-lives (*t*_1/2_) of the enzyme were calculated from the equation *t*_1__/2_ = 0.693·*k*_D_^–1^.

### Expression, purification and enzyme assay of *Gbst*-AcK

*Gbst*-AcK was expressed using the same method as *Klm*-NRK. After centrifugation, 0.5 g of recombinant *Gbst*-AcK cells were resuspended in 10 mL KPB (0.1 M, pH 7.0) and disrupted by ultrasonication. *Gbst*-AcK was purified using the same method as *Klm*-NRK. *Gbst*-AcK activity was assayed as the generation of ATP from ADP with a sufficient amount of AcP. The 1-mL reaction mixture contained 6 mM ADP, 30 mM AcP, 5 mM MgCl_2_, 50 mM pH 7.0 KPB and 20 μL enzyme solution. The solution was incubated in water bath at 30 °C for 5 min, and then 200 μL 1.0 M HCl was added for enzyme deactivation, 2.8 mL 100 mM pH 7.0 KPB was then added for dilution before high-performance liquid chromatography analysis. The ATP and ADP concentrations were measured with an HPLC (LC2010A, Shimadzu) equipped with a ChromCore C18 column (4.6 mm × 250 mm, 5-μm particle size, Nanomicrotech Co.). The mobile phase consisted of 75% solvent A (40 mM KH_2_PO_4_ and 5 mM tetramethylammonium hydrogen sulfate, and pH was adjusted to 6.2 with 1.0 M KOH) and 25% of solvent B (methanol) at a flow rate of 0.5 mL·min^–1^ and column temperature of 30 °C. The injection volume was 10 μL and individual peak areas were detected at wavelength of 254 nm. One unit of AcK was defined as the amount of the enzyme required for catalyzing the formation of 1 μmol of ATP per minute under the standard assay conditions.

### Enzymatic synthesis of NMN

The typical phosphorylation of NR at 20-mL scale was performed as follows: after 50–100 g·L^–1^ NR and 1.4 equiv. of ATP were dissolved into 12 mL ddH_2_O, and the pH was adjusted to 7.0 with 2.0 M NaOH. Furthermore, 2 mL of *Klm*-NRK supernatant (from a lysate of 50 g_wcw_/L cells suspended in pH 7.0 KPB, totally 64 U) and 2 mM MgCl_2_ (final concentration) were added, and finally ddH_2_O was supplemented to a total volume of 20 mL. The system was stirred by magnetic agitation in a reactor with a water thermostat jacket set at 30 °C. Control experiments were conducted without adding ATP or by replacing the *Klm*-NRK supernatant with lysate of *E. coli*/pET21a.

When the exogenous ATP was replaced by an ATP regeneration system (AcK/AcP), 0.2–2.0 mM ATP (final concentration), 1.4 equiv. of AcP, and 8300 U *Gbst*-AcK cell-free extract (2 mL lysate of 50 g_wcw_/L cells suspended in pH 7.0 KPB) were added to the 20-mL reaction system. The pH was adjusted to 7.0 by automatically titrating 1.0 M NaOH. Samples (each 0.1 mL) were taken periodically for HPLC analysis.

After the reaction was completed, the reaction mixture was lyophilized and the yield of NMN was determined.

### HPLC analysis

The NR and NMN concentrations were measured with HPLC method as described above.

### Purification of NMN and NMR analysis

The reaction mixture was first passed through a membrane of 3,000 molecular weight cut-off (MWCO). Then the reaction mixture was loaded on a 500-mL column of macro-porous adsorption resin (HZ-801, donated from Huazhen Company, China) and eluted with water. The fractions containing NMN were pooled and concentrated via rotary evaporation at 37 °C under vacuum, and further lyophilized to obtain NMN as white solid powder. The product structure was verified by ^1^H NMR analysis.

## Results and discussion

### Identification of nicotinamide riboside kinase

Two bioinformatic approaches were employed to identify potentially active NRKs, including pBLAST search in NCBI database with the protein sequence of *Sc*NRK1 as a probe and protein BLAST in Uniprot database with NRK as the keyword. Five genes were chosen from 750 candidates and chemically synthesized by Genscript Co. Ltd. (Nanjing, China). All the five genes were successfully expressed in *E. coli* BL21(DE3). Then their activity in phosphorylation of NR was determined. Among them, *Klm*-NRK (uniport accession number: W0TD38) from *Kluyveromyces marxianus*, *Ct*-NRK (uniport accession number: G0RZA1) from thermophilic fungus *Chaetomium thermophilum*, and *Lt-*NRK (uniport accession number: C5DCS5) from *Lachancea thermotolerans*, showed relatively higher NRK activities. Especially, *Klm*-NRK, sharing 55.1% amino acid identity with *Sc*NRK1, showed the highest activity of 632 U·g^–1^ wet cell, which was much higher than 81.7 U·g^–1^ of *Ct*-NRK and 77.8 U·g^–1^ of *Lt*-NRK. Therefore, *Klm*-NRK was chosen for further study.

### Biochemical characterization of *Klm*-NRK

The kinase *Klm*-NRK was purified to homogeneity using a Ni^+^-column (Additional file 1: Fig. S5A). *Klm*-NRK migrated at around 27 kDa, corresponding to its theoretical molecular weight. The specific activity of the purified enzyme measured under the standard condition was 7.9 U·mg^–1^ protein. The specific activities of human NRK1, human NRK2 and *Saccharomyces cerevisiae* NRK1 were 0.275 U·mg^–1^, 2.32 U·mg^–1^ and 0.535 U·mg^–1^ (Bieganowski and Brenner 2004), respectively (Table 1). Among the reported NR kinases, *Klm*-NRK showed the highest specific activity.

**Table 1:** Comparison of nicotinamide riboside kinases (NRKs) from different sources

Entry	NRK	Specific activity (U·mg^–1^)	*K*_M, ATP_ (μM)	*K*_M, NR_ (μM)
1	*Human* NRK1	0.275 ± 0.177 ^a^	4.8 ± 0.3 ^b^	3.4 ± 0.5 ^b^
2	*Human* NRK2	2.32 ± 0.20 ^a^	250 ± 12 ^b^	46 ± 8 ^b^
3	*Sc*-NRK1	0.535 ± 0.600 ^a^	NA	NA
4	*Klm*-NRK	7.90 ± 0.42 ^c^	70 ± 6 ^c^	45 ± 11 ^c^

*NA* not available, *Sc Saccharomyces cerevisiae,* *Klm* *Kluyveromyces marxianus*

^a^ Bieganowski and Brenner, 2004

^b^Dölle and Ziegler, 2009

^c^This work

**Fig. 1:**
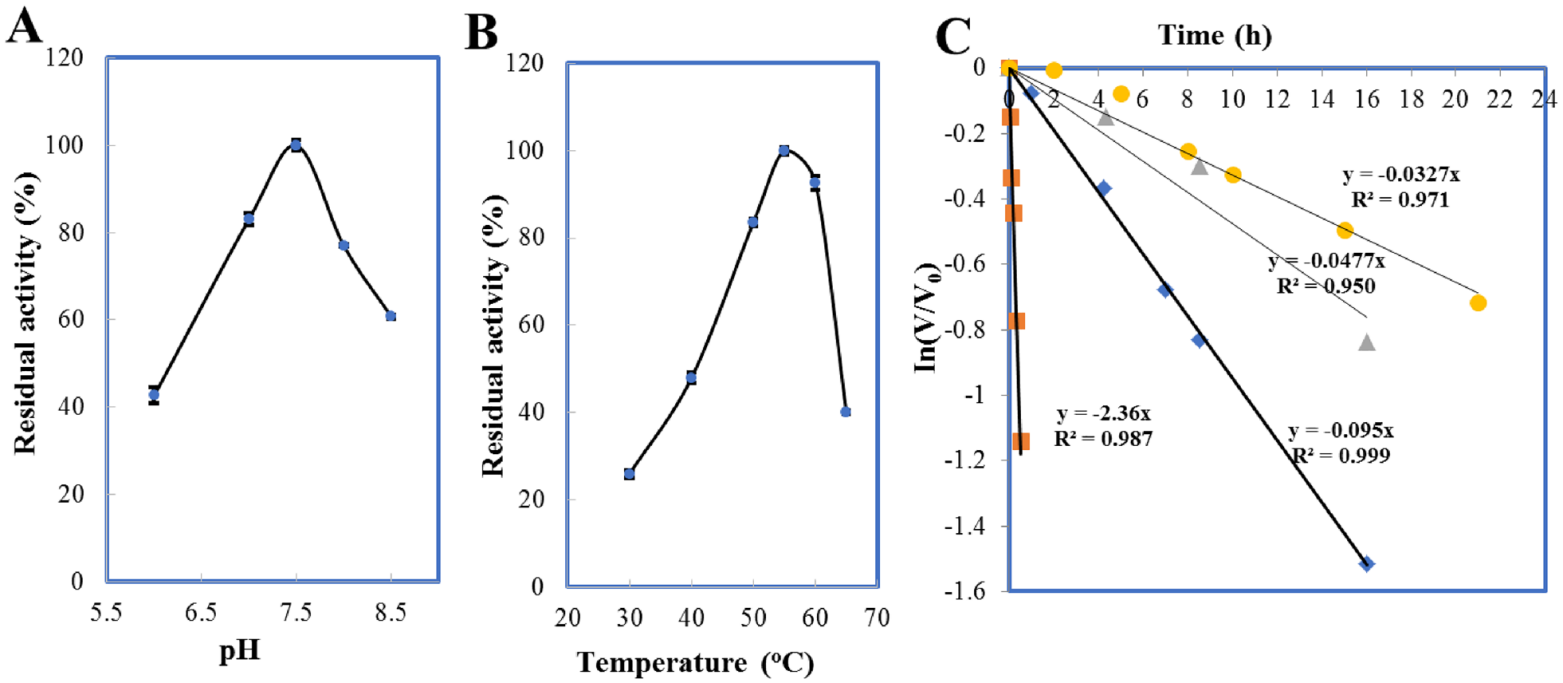
Characterization of the nicotinamide riboside kinase from *Kluyveromyces marxianus*. **A** pH optima of the purified *Klm*-NRK. The activity was measured in the potassium phosphate buffers (pH 6.0–8.5). Relative activity was expressed as a percentage of maximum activity under the experimental conditions. The maximum activity of *Klm*-NRK at pH 7.5 was 8.9 U·mg^–1^ (100%). **B** Activity–temperature profile. It was determined at various temperatures (30–65 °C) in potassium phosphate buffer (100 mM, pH 7.0). The maximum activity of *Klm*-NRK at 55 °C was 30.3 U·mg^–1^ (100%). **C **Thermal inactivation of *Klm*-NRK. Purified *Klm*-NRK (2 mg·mL^–1^) was preincubated in potassium phosphate buffer (100 mM, pH 7.0) at 30 °C (●), 40 °C (▲), 50 °C (◆) or 60 °C (■), then the residual activity was measured. The initial activity of *Klm*-NRK was 7.9 U·mg^–1^ at 30 °C (100%)

Effect of pH on the activity of *Klm*-NRK revealed that the optimal pH of *Klm*-NRK was at pH 7.5 in KPB (Fig. 1A). *Klm*-NRK displayed the highest activity at 55 °C according to the temperature profile (Fig. 1B). The purified *Klm*-NRK retained 50% of the initial activity after incubation at 30 °C for 21.2 h, 40 °C for 14.5 h, 50 °C for 7.29 h and 60 °C for 0.29 h (Fig. 1C). It can be seen that *Klm*-NRK lost half of its activity in 21.2 h even incubated at temperature as low as 30 °C, suggesting *Klm*-NRK was vulnerable. Bovine serum albumin (BSA) has been proved to be helpful for stabilization of human NRK1 at 4 °C (Sasiak and Saunders 1996). However, when 0.4 mg·mL^–1^ BSA (final concentration) was added to the solution of purified *Klm*-NRK, no obvious improvement on thermostability was observed. It is unacceptable to add potential enzyme stabilizer to NR phosphorylation reaction systems, and protein engineering and immobilization techniques will be more promising to improve the thermal stability of *Klm*-NRK.

**Fig. 2:**
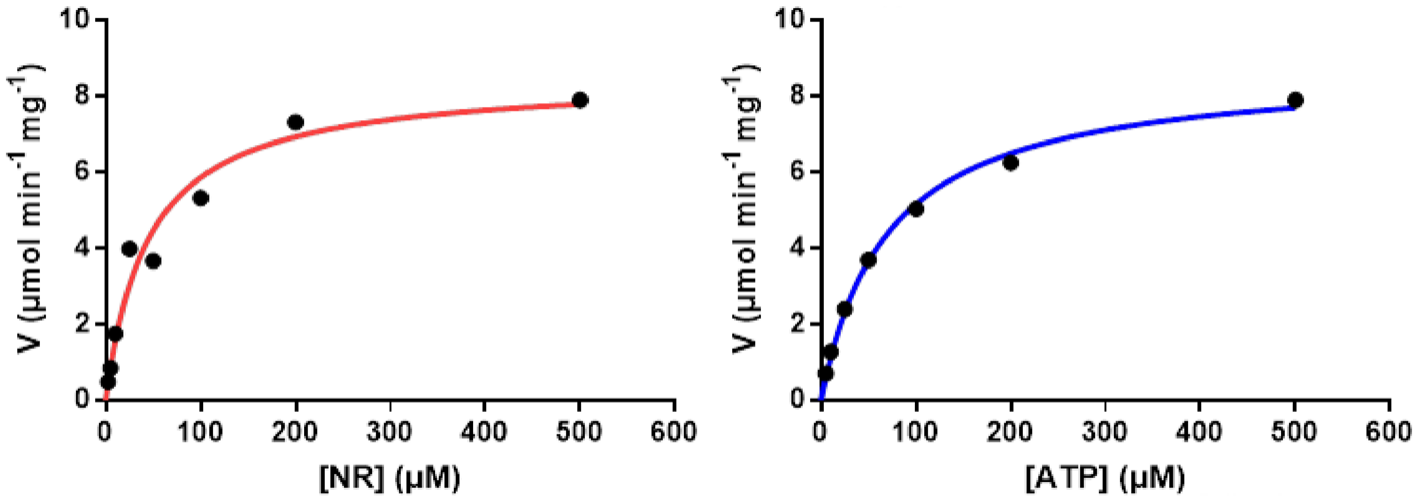
Michaelis–Menten kinetics of *Klm*-NRK for the substrates NR and ATP. Apparent parameters were determined by nonlinear regression using GraphPad Prism 7

Kinetic parameters were also determined. The *K*_M_ and *V*_max_ values of purified *Klm*-NRK measured toward ATP were 0.07 mM and 8.77 µmol·min^−1^·mg^−1^, respectively, while those measured toward NR were 0.045 mM and 8.48 µmol·min^−1^·mg^−1^. The catalytic efficiency (*k*_cat_/*K*_M_) toward ATP was 57.4 s^−1^·mM^−1^ (Fig. 2), while (*k*_cat_/*K*_M_)_NR_ toward NR was 84.4 s^−1^·mM^−1^. The catalytic efficiency of human NRK1 reported toward ATP was 6.8 s^−1^·mM^−1^ while that of human NRK2 toward ATP was 3.9 s^−1^·mM^−1^ (Tempel W et al. 2007). Therefore, the catalytic efficiency of *Klm*-NRK was 8.4-fold and 14.7-fold higher than those of human NRK1 and NRK2, respectively. All the above proved that this newly identified *Klm*-NRK is a robust enzyme with high catalytic efficiency in the phosphorylation of NR.

### Enzymatic synthesis of NMN

To utilize *Klm*-NRK for practical synthesis of NMN, the reaction conditions were optimized. An excess amount (1.4 eq.) of ATP was first used as the phosphoryl donor. As shown in Table 2, 99.2% conversion of NR was achieved within 10 h in the presence of 64 U *Klm*-NRK at substrate loads of 50 g·L^–1^ NR and 141 g·L^–1^ ATP (Table 2, entry 1). When the NR load was increased up to 75 g·L^–1^, it could also be almost completely transformed under the same condition within 27 h (Table 2, entry 2). However, when the NR load was further elevated up to 100 g·L^–1^, only 82.1% conversion was achieved at 27 h (Table 2, entry 3). This might be attributed to the high loads of ATP that might influence the activity of *Klm*-NRK.

**Table 2:** Enzymatic phosphorylation of nicotinamide riboside with *Klm*-NRK


Entry	NR (g·L^–1^)	ATP (g·L^–1^)	P_*i*_ donor	Time (h)	Conv. (%)
1	50	141	ATP	8	99.2
2	75	211	ATP	27	99.2
3	100	282	ATP	27	82.1
4	50	0.114	AcP/AcK	6	99.7
5	50	1.14	AcP/AcK	2	99.7
6	100	1.14	AcP/AcK	8	98.3

The main drawbacks of using high concentration of externally added ATP in the reaction include both the high cost of ATP and the downstream processing issue caused by the formation of an equal mole of byproduct ADP (Additional file 1: Fig. S7). Therefore, an ATP regeneration system (AcK/AcP) was then introduced to replace the exogenously added ATP in high concentration. Consequently, even if only 0.2 mM ATP was added exogenously, an almost complete conversion of 50 g·L^–1^ NR was easily achieved within 6 h in the presence of 64 U *Klm*-NRK and 8300 U *Gbst*-AcK (Table 2, entry 4). Because the *K*_M_ values of *Gbst*-AcK measured towards ATP and ADP were 1.2 mM and 0.8 mM (Nakajima et al. 1978), a higher concentration (2 mM) of initially loaded ATP was then investigated, resulting in a complete conversion of 50 g·L^–1^ NR into NMN within only 2 h (Table 2, entry 5). Finally, when the NR loading was further escalated to 100 g·L^–1^, 98.3% conversion was achieved in 8 h (Table 2, entry 6), affording 1.87 g NMN (84.2% molar yield) which was isolated from the lyophilized crude product (*ca.* 3.77 g), representing a space–time yield of 281 g L^−1^ d^−1^.

ATP was recycled approximately 144.8 times in the system since only 2 mM ATP was initially added, avoiding inhibitory effect of the coproduct ADP. The extremely high specific activity of *Gbst*-AcK (1876 U·mg^–1^), relatively low molecular weight (43.4 kDa, Additional file 1: Fig. S5B) and the high substrate loading contribute to a very high total turnover number of 56,876 mol product per mol *Gbst*-AcK, indicating its great potential for ATP regeneration.

The result of control experiments with neither ATP nor the enzyme (*Klm*-NRK) proved that no NR was converted. However, even when the lysate of blank *E. coli/*pET21a cells was added, approximately 3.5% of NR decomposition was still monitored in 8 h, suggesting a detectable activity of nucleotide phosphorylase inside the *E. coli* BL21(DE3) cells. Therefore, a better host with less nucleotide phosphorylase activity may further increase the product yield.

Totally 750 mL water was used for the purification of NMN during the column chromatography with a macro-porous adsorption resin (bed volume: 500 mL), among which 250 mL water was used to pre-wash the impurities before elution. Subsequently, the NMN-containing eluent (*ca.* 500 mL) was concentrated to a final volume of about 50 mL. Furthermore, the concentrate was lyophilized, affording 1.24 g NMN in > 97% purity. ^1^H-NMR (400 MHz, D_2_O), δ/ppm: 9.47 (s, 1H), 9.29 (d, *J* = 6.2 Hz, 1H), 8.99 (d, *J* = 8.1 Hz, 1H), 8.35–8.26 (m, 1H), 6.22 (d, *J* = 5.4 Hz, 1H), 4.65 (s, 1H), 4.57 (t, *J* = 5.2 Hz, 1H), 4.45 (dd, *J* = 4.9, 2.5 Hz, 1H), 4.31 (d, *J* = 12.0 Hz, 1H), 4.15 (d, *J* = 9.1 Hz, 1H); ^13^C-NMR (100 MHz, D_2_O), δ/ppm: 165.84, 145.99, 142.50, 139.87, 133.95, 128.53, 99.98, 87.50, 87.42, 77.75, 71.04, 64.19, 64.14.

## Conclusions

In summary, *Klm*-NRK, a nicotinamide riboside kinase with the highest activity reported so far was discovered from *Kluyveromyces marxianus*. For the first time, the enzymatic synthesis of NMN was achieved in high-titer (93.5 g·L^–1^) by employing the new and highly active NRK, and by adopting an efficient and cost-effective ATP regeneration system, which resulted in a space–time yield of 281 g·L^−1^·day^−1^. Further studies to improve the catalytic performance of *Klm-*NRK by protein engineering and immobilization are now ongoing, which are expected to make *Klm-*NRK a more efficient tool for the large-scale manufacturing of NMN.

### Supplementary Information

**Additional file 1: Figure S1**. Un-optimized Klm-nrk gene analysis. **Figure S2**. Optimized Klm-nrk gene analysis. **Figure S3**. Un-optimized Gbst-ack gene analysis. **Figure S4**. Optimized Gbst-ack gene analysis. **Figure S5**. Analysis of the purified Klm-NRK and Gbst-AcK by SDS-PAGE (12%). **Figure S6**. HPLC spectra of NR, NMN, ADP and ATP standards. **Figure S7**. Representative HPLC spectra for enzymatic phosphorylation of NR. **Figure S8**. NMR spectra of enzymatically synthesized NMN.

## Data Availability

All data generated or analyzed during this study are included in this article and its supplementary information file.

## Abbreviations

NMN: Nicotinamide mononucleotide
NR: Nicotinamide riboside
NAD^+^/NADP^+^: Nicotinamide adenine dinucleotide/nicotinamide adenine dinucleotide phosphate
TMSOTf: Trimethylsilyl trifluoromethanesulfonate
*Klm*-NRK: Nicotinamide riboside kinase from *Kluyveromyces marxianus*
*Gbst*-AcK: Acetate kinase from *Bacillus stearothermophilus*
AcP: Acetyl phosphate
ADP: Adenosine diphosphate
ATP: Adenosine triphosphate
