# Supplementary material for: Enzymatic synthesis of high-titer nicotinamide mononucleotide with a new nicotinamide riboside kinase and an efficient ATP regeneration system
Source: Bioresour Bioprocess. 2022 Mar 21;9(1):26. doi: 10.1186/s40643-022-00514-6 (PMC10992250; doi:10.1186/s40643-022-00514-6)
Supplement: Supplementary file 1 — Additional file 1: Figure S1. Un-optimized Klm-nrk gene analysis. Figure S2. Optimized Klm-nrk gene analysis. Figure S3. Un-optimized Gbst-ack gene analysis. Figure S4. Optimized Gbst-ack gene analysis. Figure S5. Analysis of the purified Klm-NRK and Gbst-AcK by SDS-PAGE (12%). Figure S6. HPLC spectra of NR, NMN, ADP and ATP standards. Figure S7. Representative HPLC spectra for enzymatic phosphorylation of NR. Figure S8. NMR spectra of enzymatically synthesized NMN. [file 40643_2022_514_MOESM1_ESM.docx]

*Supporting Information*

*for*

**Enzymatic synthesis of high-titer nicotinamide mononucleotide with a new nicotinamide riboside kinase and an efficient ATP regeneration system**

Xiao-Long Qian, Yi-Si Dai, Chun-Xiu Li, Jiang Pan, Jian-He Xu*

State Key Laboratory of Bioreactor Engineering, Shanghai Collaborative Innovation Center for Biomanufacturing, East China University of Science and Technology, Shanghai 200237, China. E-mail: jianhexu@ecust.edu.cn

**Table of Contents**

1. [**Sequence Optimization of *Klm-nrk and Gbst-ack* Genes** S3](#_Toc87734755)

**2.** [**SDS-PAGE of Purified Enzyme *Klm*-NRK and *Gbst*-AcK** S10](#_Toc87734756)

**3.** [**HPLC spectra of NR, NMN, ADP and ATP** S11](#_Toc87734757)

**4.** **^1^H- and ^13^C-**[**NMR spectra of synthesized NMN** S13](#_Toc87734758)

# **Sequence Optimization of *Klm*-*nrk* and *Gbst*-*ack* Genes**

The *Klm*-*nrk* and *Gbst*-*ack* genes were analyzed by an online software, **graphical codon usage analyser** (<http://gcua.schoedl.de/sequential_v2.html>). Codon usage bias adjustment and GC content adjustment were used for better expression in *E. coli.*

NRK_Klm

Amino acid sequence:

>tr|W0TD38|W0TD38_KLUMD Nicotinamide riboside kinase OS=*Kluyveromyces* *marxianus* (strain DMKU3-1042 / BCC 29191 / NBRC 104275) OX=1003335 GN=NRK1 PE=4 SV=1

MTTTKVKLIAISGCSSSGKTTLAKFLANAIPGCILIHEDDFYKPDSEIPINEKYGVADWDCPEALDLDAFKRELDLIKTTGSIKTKLIHNENVDDIGKFNIKQEDWDALRAKLSSVIESDLKVVLVDGFMIFNDEELMRKFDIRIFVRAPYEVLSRRRHARAGYKTLESFWVDPPYYFDEFVYRAYREEHKHLFVNEDVEGSLRSDAGLFELINDDETEITTALNTIADYIVSHLDAN

Original gene sequence:

>ENA|BAO41552|BAO41552.1 *Kluyveromyces marxianus* DMKU3-1042 nicotinamide riboside kinase

ATGACCACAACAAAGGTAAAACTCATTGCAATAAGCGGTTGTTCGTCCTCGGGAAAAACAACGCTTGCCAAGTTCCTTGCTAATGCTATACCGGGCTGCATACTTATACATGAGGACGACTTCTATAAACCGGATTCTGAGATTCCTATTAACGAAAAATACGGCGTAGCTGATTGGGACTGTCCAGAAGCACTGGATCTGGATGCATTCAAAAGAGAACTTGACCTAATAAAAACTACTGGTTCCATTAAGACGAAGTTGATTCACAATGAAAATGTGGATGATATTGGTAAATTCAACATTAAACAAGAAGATTGGGATGCCTTAAGAGCAAAACTCTCATCGGTTATTGAATCTGACTTGAAAGTAGTGCTTGTCGATGGTTTCATGATTTTCAATGATGAGGAATTAATGAGGAAGTTTGACATTAGAATATTTGTGCGTGCACCATACGAGGTTTTAAGCAGAAGACGTCACGCTAGAGCTGGTTACAAAACGTTAGAATCTTTTTGGGTTGACCCTCCATACTACTTCGATGAATTTGTATACAGGGCTTACAGAGAGGAGCATAAACACTTATTTGTAAATGAAGACGTCGAAGGTAGTTTAAGGTCCGATGCAGGGTTATTCGAACTAATTAATGACGACGAAACTGAAATTACTACAGCATTGAATACAATCGCCGATTACATCGTCTCTCATTTAGACGCAAACTAG


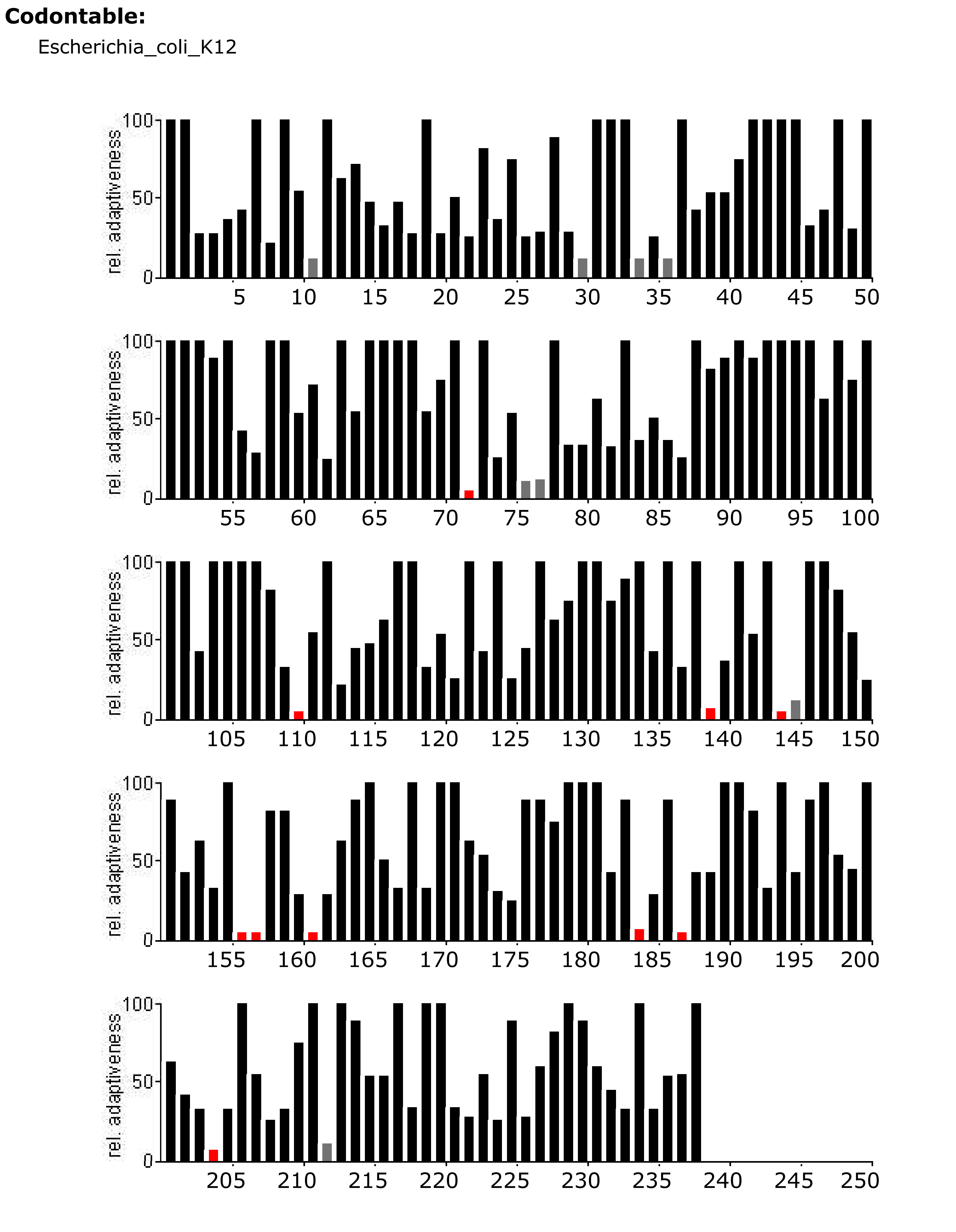


Figure S1. Un-optimized *Klm*-nrk gene analysis.

**The optimized gene sequence of *Klm*-nrk**:

*CATATG*ACCACCACCAAGGTGAAACTGATCGCGATTAGCGGTTGCAGCAGCAGCGGCAAGACCACCCTGGCGAAATTCCTGGCGAACGCGATCCCGGGTTGCATCCTGATTCACGAGGACGATTTTTACAAGCCGGATAGCGAGATCCCGATTAACGAAAAATATGGTGTGGCGGACTGGGATTGCCCGGAGGCGCTGGACCTGGATGCGTTCAAGCGTGAACTGGACCTGATCAAAACCACCGGTAGCATCAAGACCAAACTGATTCACAACGAGAACGTTGACGATATCGGCAAGTTTAACATTAAACAGGAAGACTGGGATGCGCTGCGTGCGAAGCTGAGCAGCGTGATCGAGAGCGACCTGAAAGTGGTTCTGGTTGATGGTTTCATGATTTTTAACGACGAGGAACTGATGCGTAAGTTCGATATCCGTATTTTTGTGCGTGCGCCGTACGAAGTTCTGAGCCGTCGTCGTCACGCGCGTGCGGGTTATAAAACCCTGGAGAGCTTCTGGGTGGACCCGCCGTACTATTTCGATGAATTTGTTTACCGTGCGTATCGTGAGGAACACAAGCACCTGTTCGTGAACGAGGATGTTGAAGGTAGCCTGCGTAGCGACGCGGGCCTGTTTGAACTGATCAACGACGATGAGACCGAAATTACCACCGCGCTGAACACCATCGCGGATTACATTGTTAGCCACCTGGACGCGAAC*CTCGAG*


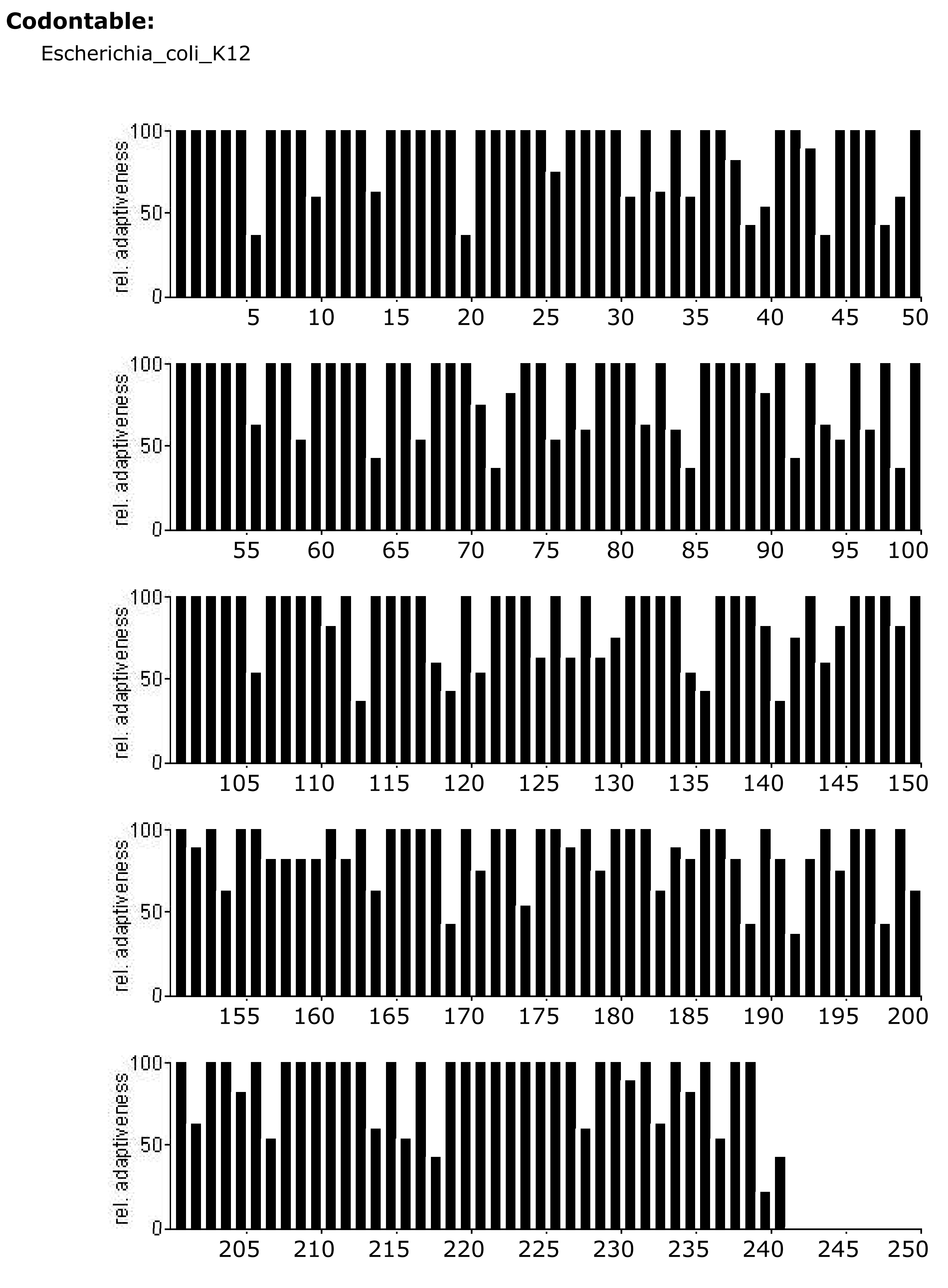


Figure S2. Optimized *Klm*-nrk gene analysis.

***Gbst*-ack gene**

Amino acid sequence:

>tr|A0A0K9HLE0|A0A0K9HLE0_GEOSE Acetate kinase OS=*Geobacillus* *stearothermophilus* OX=1422 GN=ackA PE=3 SV=1

MAKVLAVNAGSSSLKFQLFDMPAETVLTKGIVERIGFDDAIFTIVVNGEKQREVTSIPNHAVAVKLLLDKLIRYGIIRSFDEIDGIGHRVVHGGEKFSDSVLITDEVIKQIEEVSELAPLHNPANLVGIRAFQEVLPNVPAVAVFDTAFHQTMPEQSFLYSLPYEYYTKFGIRKYGFHGTSHKYVTQRAAELLGRPIEQLRLISCHLGNGASIAAVEGGKSIDTSMGFTPLAGVAMGTRSGNIDPALIPYIMEKTGMTVNEVIEVLNKKSGMLGISGISSDLRDLEKAAAEGNERAELALEVFANRIHKYIGSYAARMCGVDAIIFTAGIGENSEVVRAKVLRGLEFMGVYWDPILNKVRGKEAFISYPHSPVKVLVIPTNEEVMIARDVMRLANL

**Original gene sequence of *Gbst*-ack**

>ENA|KYD23390|KYD23390.1 *Geobacillus stearothermophilus* Acetate kinase

ATGGCAAAAGTGTTAGCCGTTAATGCGGGAAGTTCTTCGTTGAAATTCCAATTGTTTGACATGCCGGCGGAAACGGTGTTAACGAAAGGAATCGTCGAGCGGATCGGCTTTGACGACGCGATTTTTACGATCGTCGTGAACGGGGAGAAACAGCGGGAAGTCACTTCCATCCCGAACCATGCCGTGGCGGTGAAACTGCTGCTTGACAAACTGATTCGCTATGGCATCATCCGGTCATTTGACGAAATTGACGGCATCGGCCATCGCGTCGTCCACGGCGGGGAGAAGTTCAGCGATTCGGTGTTGATCACCGATGAGGTGATAAAACAAATCGAAGAAGTGTCCGAGCTCGCTCCGCTTCATAACCCGGCCAACCTCGTCGGCATCCGCGCGTTTCAGGAAGTGCTGCCGAACGTGCCGGCCGTCGCCGTTTTTGATACGGCGTTTCACCAAACGATGCCGGAACAGTCGTTTTTGTACAGCTTGCCGTATGAGTATTACACGAAATTCGGCATTCGCAAGTACGGCTTCCATGGCACGTCGCACAAATACGTCACCCAGCGGGCGGCGGAGCTTCTCGGCCGGCCGATCGAGCAGCTGCGCCTCATCTCGTGCCATTTAGGCAACGGGGCGAGCATCGCGGCGGTCGAAGGCGGCAAATCGATCGACACGTCGATGGGCTTTACGCCATTAGCGGGCGTCGCGATGGGGACGCGCTCTGGCAACATCGATCCGGCGCTTATCCCATACATTATGGAAAAAACAGGAATGACCGTTAATGAAGTGATTGAAGTGCTGAATAAAAAGAGCGGCATGCTCGGCATCTCCGGCATCTCGAGCGACTTGCGCGACTTGGAAAAAGCGGCCGCCGAAGGAAATGAGCGCGCGGAACTTGCGTTGGAAGTGTTTGCGAACCGCATTCATAAATACATCGGCTCGTATGCGGCGCGCATGTGCGGCGTCGACGCCATCATTTTCACCGCCGGCATCGGCGAAAACAGCGAAGTCGTGCGGGCCAAAGTGCTGCGCGGCCTCGAGTTTATGGGAGTTTACTGGGATCCCATCCTAAACAAAGTGCGCGGCAAAGAAGCGTTCATCAGCTACCCGCACTCGCCGGTCAAAGTGCTCGTCATCCCGACGAACGAAGAGGTCATGATCGCCCGTGATGTCATGCGGCTGGCGAATTTGTAA


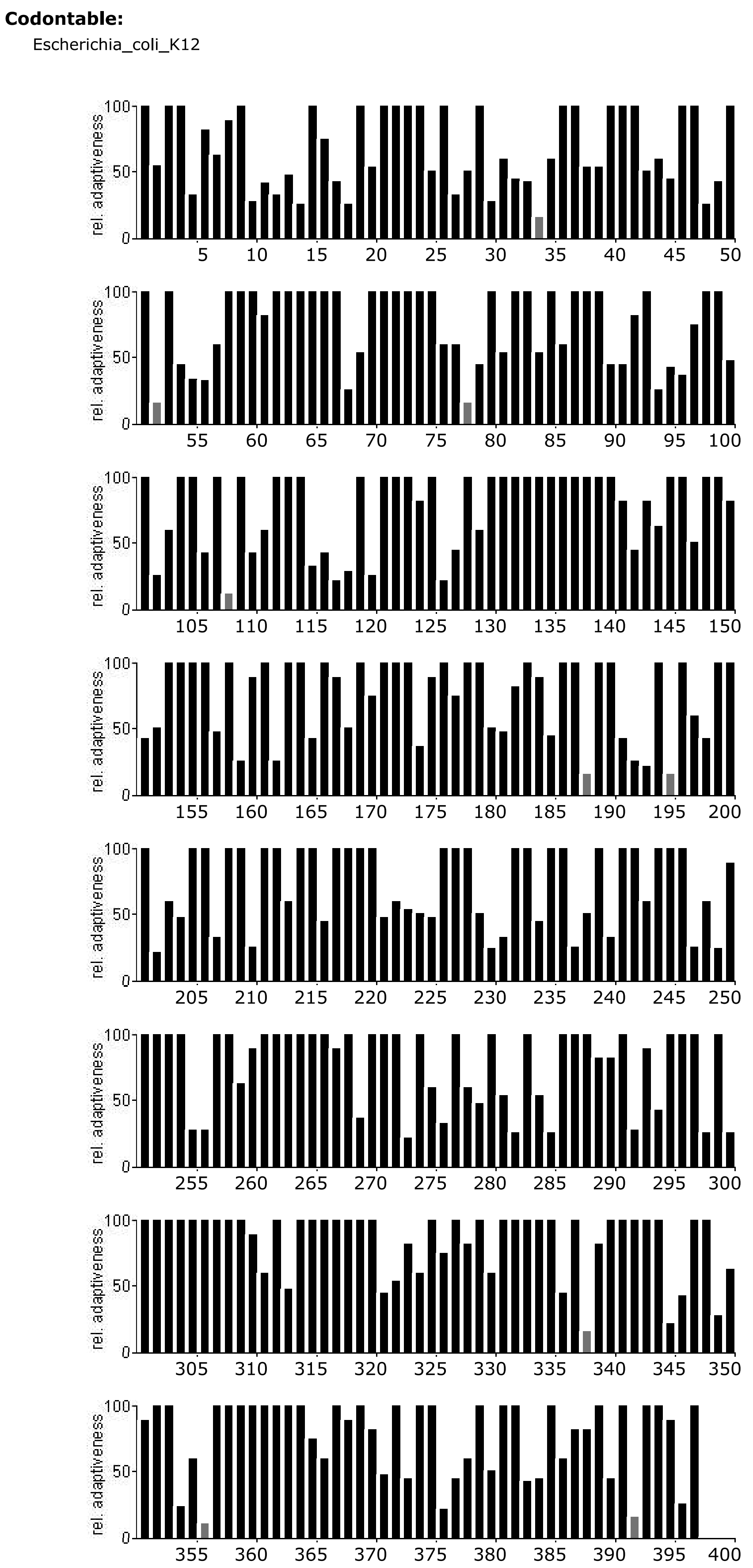


Figure S3. Un-optimized *Gbst*-ack gene analysis.

**Optimized gene sequence of *Gbst*-ack**

*CATATG*GCGAAGGTTCTGGCGGTTAATGCGGGTAGCAGCAGCCTGAAATTCCAACTGTTTGACATGCCGGCGGAGACCGTTCTGACCAAGGGTATCGTGGAACGTATTGGCTTCGACGATGCGATCTTTACCATTGTGGTTAACGGTGAAAAGCAGCGTGAGGTTACCAGCATCCCGAACCACGCGGTGGCGGTTAAGCTGCTGCTGGACAAACTGATTCGTTACGGCATCATTCGTAGCTTCGACGAAATCGATGGTATTGGCCACCGTGTGGTTCACGGTGGCGAGAAGTTTAGCGACAGCGTTCTGATCACCGATGAAGTGATCAAACAGATTGAGGAAGTGAGCGAGCTGGCGCCGCTGCACAACCCGGCGAACCTGGTTGGTATTCGTGCGTTCCAAGAGGTTCTGCCGAACGTGCCGGCGGTGGCGGTTTTCGATACCGCGTTTCACCAGACCATGCCGGAACAAAGCTTTCTGTACAGCCTGCCGTATGAGTACTATACCAAGTTCGGTATCCGTAAATACGGTTTTCACGGCACCAGCCACAAATATGTTACCCAGCGTGCGGCGGAACTGCTGGGCCGTCCGATTGAGCAACTGCGTCTGATTAGCTGCCATCTGGGTAACGGTGCGAGCATTGCGGCGGTGGAAGGTGGCAAGAGCATTGACACCAGCATGGGTTTCACCCCGCTGGCGGGTGTTGCGATGGGTACCCGTAGCGGCAACATCGATCCGGCGCTGATCCCGTATATTATGGAGAAAACCGGCATGACCGTGAACGAGGTTATTGAAGTGCTGAACAAGAAAAGCGGTATGCTGGGCATCAGCGGTATTAGCAGCGACCTGCGTGATCTGGAAAAAGCGGCGGCGGAGGGTAACGAACGTGCGGAGCTGGCGCTGGAGGTGTTTGCGAACCGTATCCACAAATACATTGGTAGCTATGCGGCGCGTATGTGCGGCGTTGACGCGATCATTTTTACCGCGGGTATCGGCGAAAACAGCGAGGTGGTTCGTGCGAAGGTTCTGCGTGGTCTGGAATTCATGGGCGTGTACTGGGATCCGATCCTGAACAAGGTTCGTGGTAAAGAGGCGTTTATTAGCTATCCGCACAGCCCGGTGAAAGTTCTGGTTATTCCGACCAACGAAGAAGTGATGATTGCGCGTGATGTTATGCGTCTGGCGAACCTG*CTCGAG*


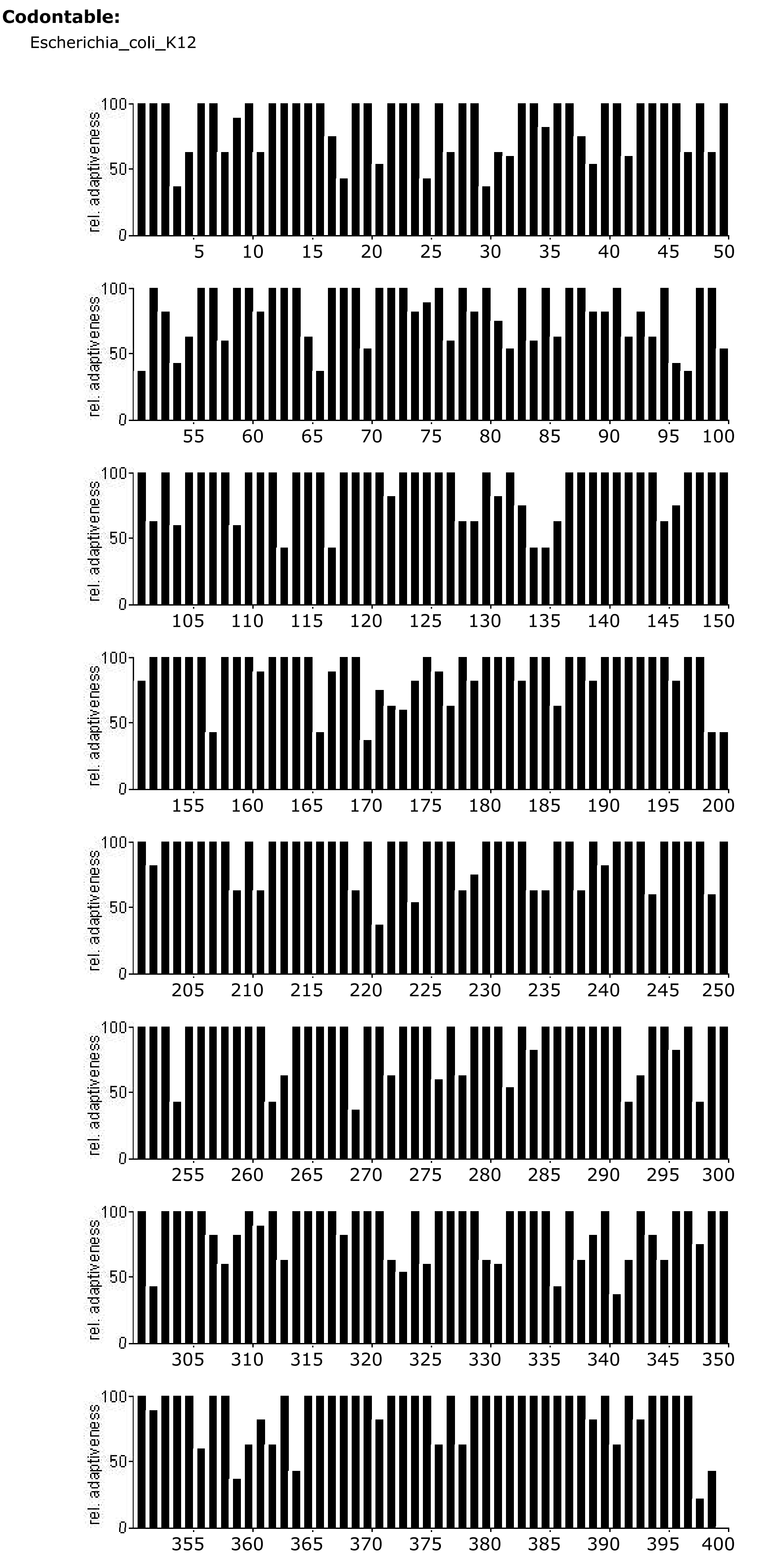


Figure S4. Optimized *Gbst*-ack gene analysis.

1. [**SDS-PAGE of purified *Klm*-NRK**](#_Toc87734756) **and *Gbst*-AcK**


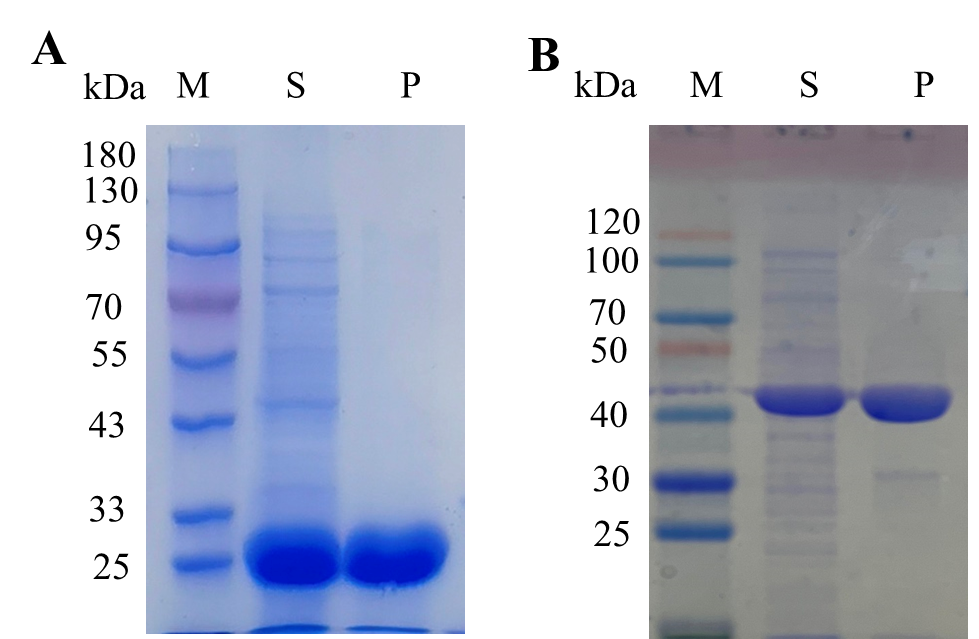


# **Figure S5.** **Analysis of the purified *Klm*-NRK and *Gbst*-AcK by SDS-PAGE (12%).**

**A:** (**M**) Protein marker; (**S**) Cell free extract of the recombinantly expressed *Klm*-NRK;

(**P**) Purified *Klm*-NRK.

**B:** (**M**) Protein marker; (**S**) Cell free extract of the recombinantly expressed *Gbst*-AcK;

(**P**) Purified *Gbst*-AcK.

1. **HPLC spectra of NR, NMN, ADP and ATP**


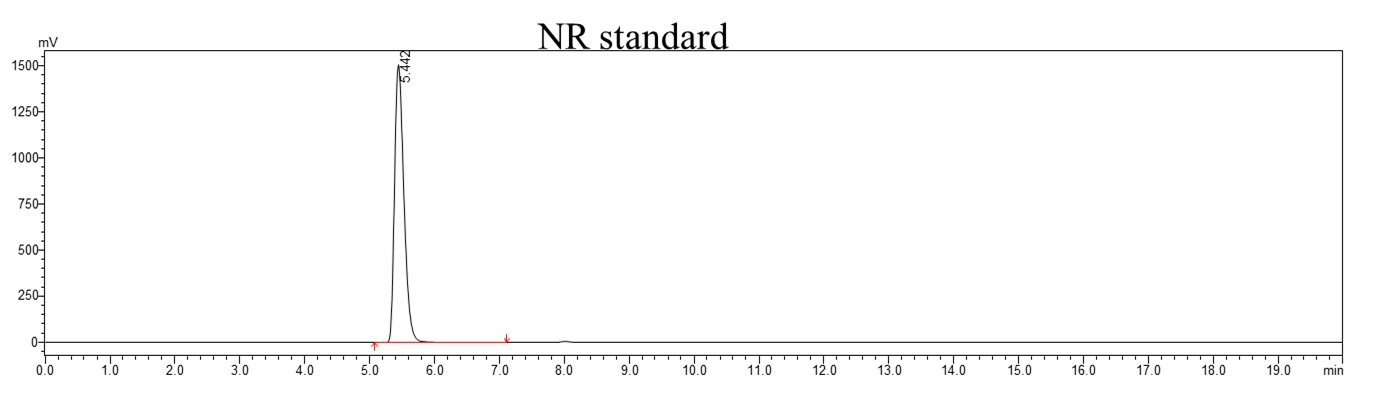


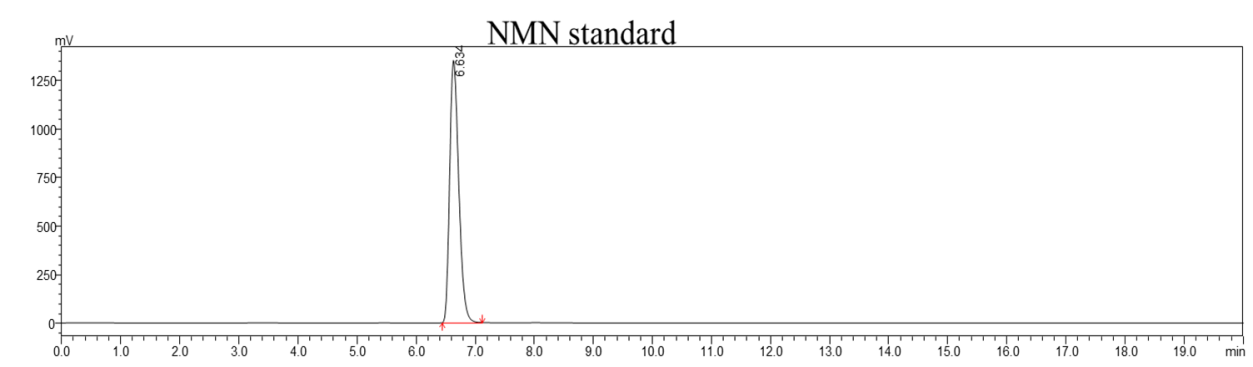


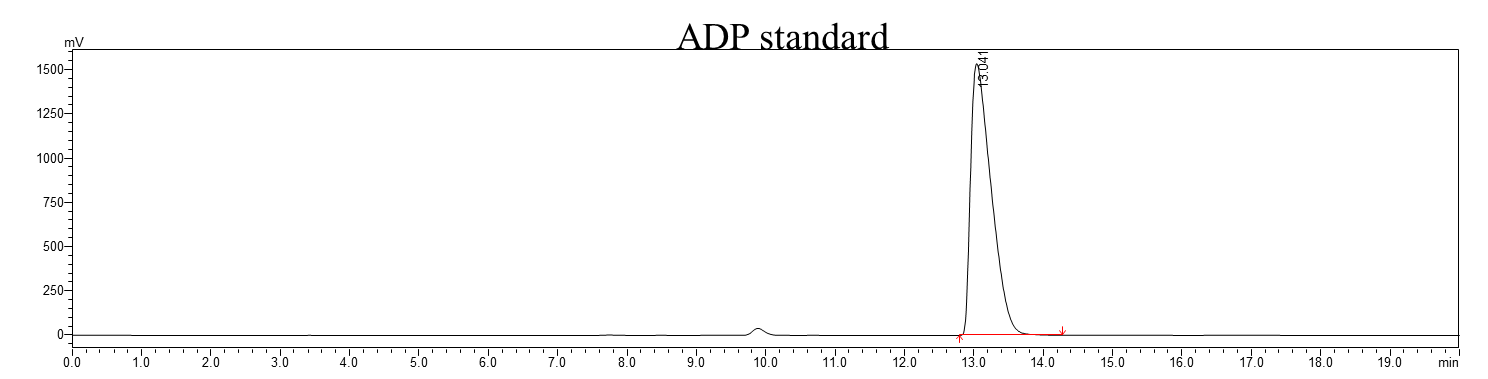


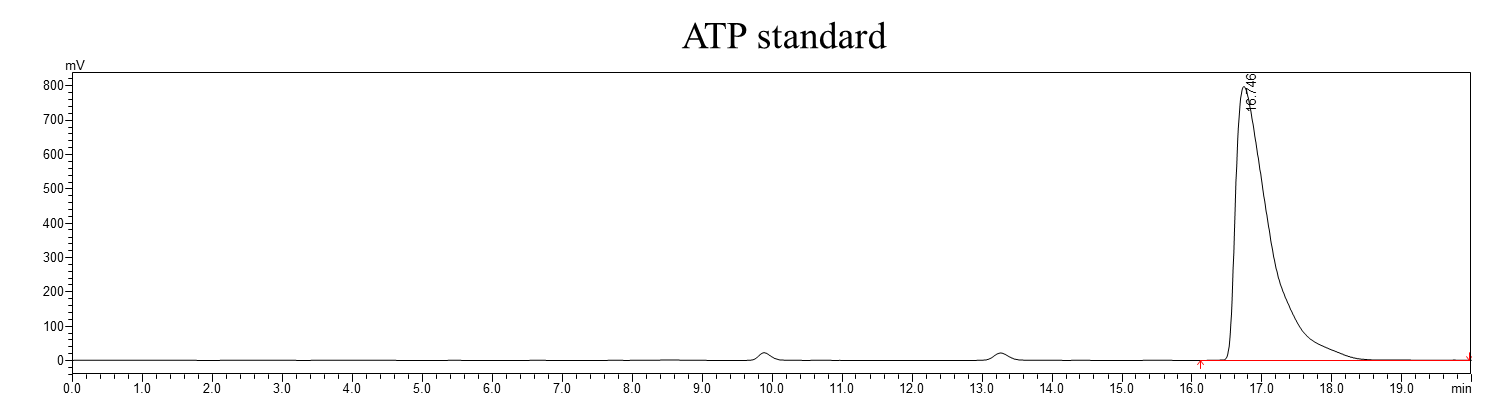


Figure S6. HPLC spectra of NR, NMN, ADP and ATP standards.

Retention time: NR 5.44 min; NMN 6.63 min; ADP 13.04 min; ATP 16.75 min.


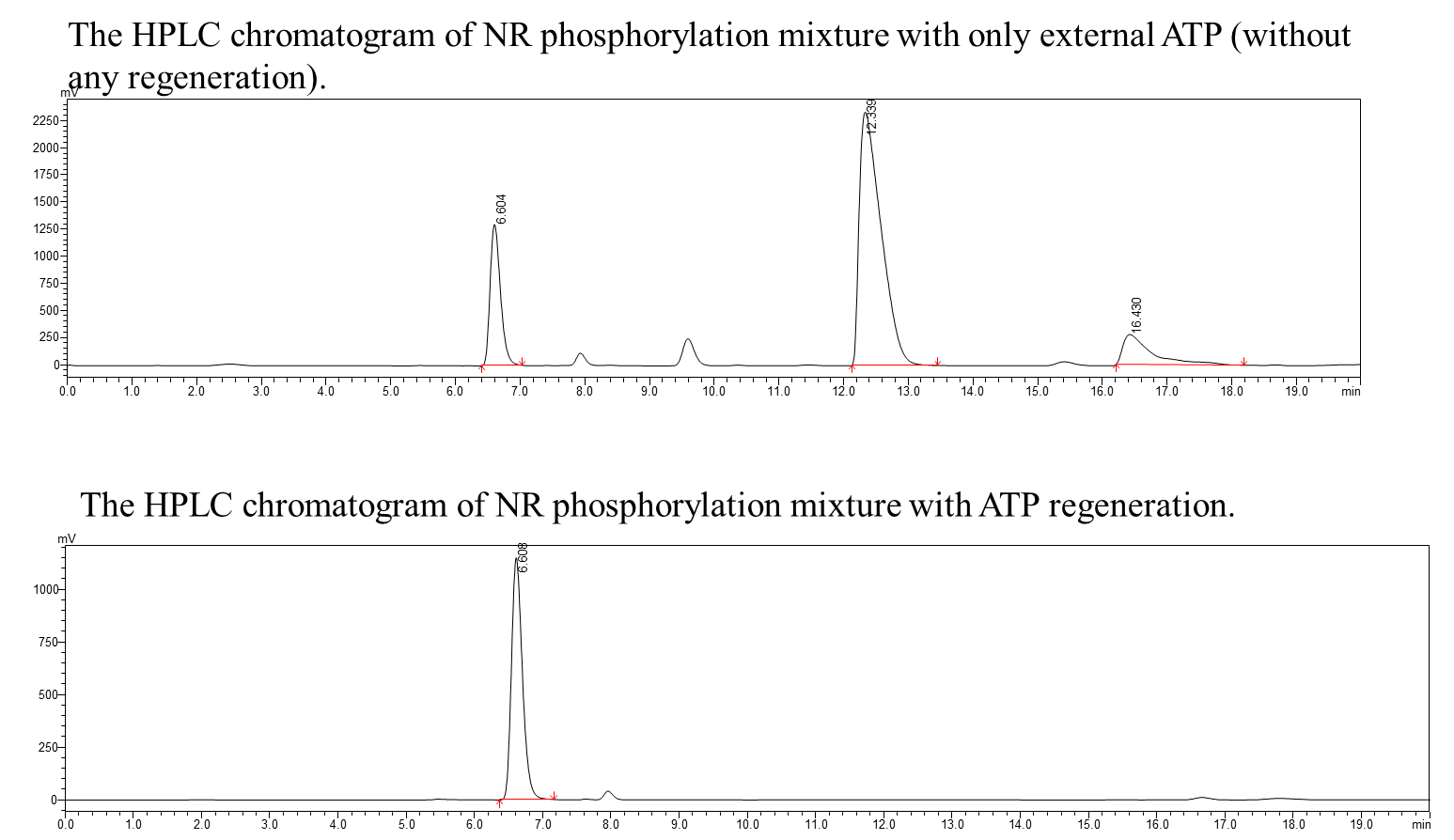


Figure S7. Representative HPLC spectra for enzymatic phosphorylation of NR.

The enzymatic phosphorylation reactions of NR were compared, with or without ATP regeneration.

**4. ^1^H- and ^13^C-NMR spectra of synthesized NMN**


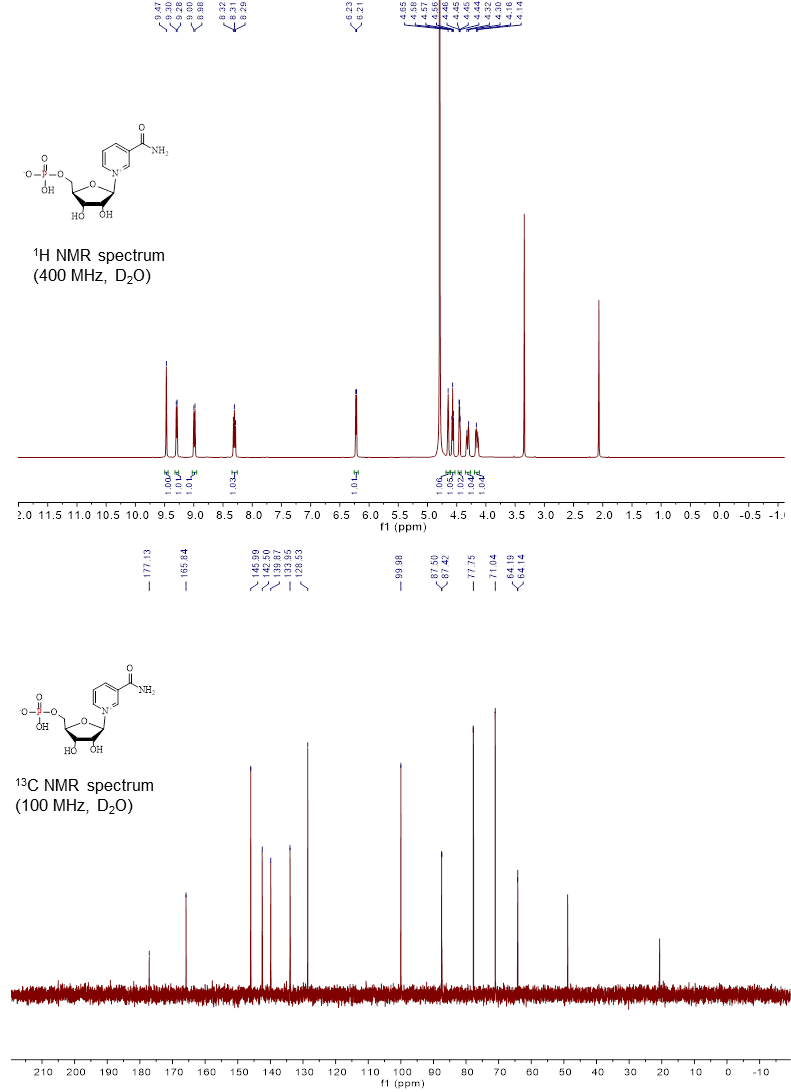


**Figure S8. NMR spectra of enzymatically synthesized NMN.**
